# dbAMP: an integrated resource for exploring antimicrobial peptides with functional activities and physicochemical properties on transcriptome and proteome data

**DOI:** 10.1093/nar/gky1030

**Published:** 2018-10-31

**Authors:** Jhih-Hua Jhong, Yu-Hsiang Chi, Wen-Chi Li, Tsai-Hsuan Lin, Kai-Yao Huang, Tzong-Yi Lee

**Affiliations:** 1 Department of Computer Science and Engineering, Yuan Ze University, Taoyuan 320, Taiwan; 2 School of Science and Engineering, The Chinese University of Hong Kong, Shenzhen 518172, China; 3 Warshel Institute for Computational Biology, The Chinese University of Hong Kong, Shenzhen 518172, China

## Abstract

Antimicrobial peptides (AMPs), naturally encoded from genes and generally contained 10–100 amino acids, are crucial components of the innate immune system and can protect the host from various pathogenic bacteria, as well as viruses. In recent years, the widespread use of antibiotics has inspired the rapid growth of antibiotic-resistant microorganisms that usually induce critical infection and pathogenesis. An increasing interest therefore was motivated to explore natural AMPs that enable the development of new antibiotics. With the potential of AMPs being as new drugs for multidrug-resistant pathogens, we were thus motivated to develop a database (dbAMP, http://csb.cse.yzu.edu.tw/dbAMP/) by accumulating comprehensive AMPs from public domain and manually curating literature. Currently in dbAMP there are 12 389 unique entries, including 4271 experimentally verified AMPs and 8118 putative AMPs along with their functional activities, supported by 1924 research articles. The advent of high-throughput biotechnologies, such as mass spectrometry and next-generation sequencing, has led us to further expand dbAMP as a database-assisted platform for providing comprehensively functional and physicochemical analyses for AMPs based on the large-scale transcriptome and proteome data. Significant improvements available in dbAMP include the information of AMP–protein interactions, antimicrobial potency analysis for ‘cryptic’ region detection, annotations of AMP target species, as well as AMP detection on transcriptome and proteome datasets. Additionally, a Docker container has been developed as a downloadable package for discovering known and novel AMPs on high-throughput omics data. The user-friendly visualization interfaces have been created to facilitate peptide searching, browsing, and sequence alignment against dbAMP entries. All the facilities integrated into dbAMP can promote the functional analyses of AMPs and the discovery of new antimicrobial drugs.

## INTRODUCTION

Antimicrobial peptides (AMPs), naturally encoded from genes and generally contained 10–100 amino acids, are produced by different organisms as a defense mechanism against microbial invasions (1–3). AMPs are a particularly functional group of protein molecules, and most of them are α-helical and amphipathic, which means they have hydrophilic residues on one side, and hydrophobic residues on the other side. In typical, AMPs contain more positively charged residues on hydrophilic side for attaching to the membrane surface of microbes. On the other hand, the hydrophobic side allows AMPs anchoring into the membrane lipid bilayer for leading to depolarization of the membrane and to cell death by worm-hole pore, barrel-stave pore, or carpet models (4–6). AMPs can play as the first line of innate immune systems of all living organisms, ranging from prokaryotes to humans, for enabling the cell death of microbes either by disrupting its cell membrane or its intracellular functions (7,8). Multicellular hosts can thus adapt to pathogenic microbes via their innate immunity through the rapid synthesis and release of diversely short peptides known as AMPs (9). AMPs are comprehensively distributed in nature and have been isolated from a variety of sources including bacteria, plants, vertebrates, and mammals (10–12). Recently, a couple of sophisticated approaches, such as AMP mimetics (13), AMP congeners, cyclotides and stabilized AMPs, AMP conjugates, and immobilised AMPs (14), were devoted to the discovery of AMPs.

AMPs have been discovered that they have a broad spectrum of antimicrobial activities against a variety of pathogens, including not only gram-positive and gram-negative bacterial, fungi, parasite, protozoans, viruses but also insects and several sorts of tumor (15,16). In addition to their antimicrobial properties, AMPs also can function as mediators of inflammation with impact on human epithelial and inflammatory cells such as cell proliferation, immune induction, and wound healing (17). AMPs have been validated to be associated with variously functional activities. A previous study showed that a little modification on the primary sequence of AMPs may influence its specificity and activity along with structural changes (18). Therefore, a full understanding of amino acid composition of AMPs is a key to manufacturing antimicrobial agents by reducing their cytotoxicities and increasing their activities. In the last decade, AMPs are becoming the attractive drug targets in clinical applications owing to the broad spectrum of antimicrobial activities and low propensity for drug-resistant development (19). AMPs were thus attracting the attention of many investigators as a substitute for conventional antibiotics. Hence, a further investigation into the structure–activity relationship of AMPs is an urgent need to the development of new antibiotics or drugs (20).

In recent year, the widespread use of antibiotics has inspired the rapid growth of antibiotic-resistant microorganisms that usually induce critical infection and pathogenesis. An increasing interest was therefore motivated to accumulate natural AMPs with an attempt to enable the development of new antibiotics. Several AMP databases have been developed for specific species, including PenBase for shrimp (21), PhytAMP for plants (22), DADP for anurans (23), as well as BACTIBASE (24), BAGEL4 (25) and YADAMP (26) for bacterial. Additionally, a couple of resources have integrated a broad spectrum of AMPs on multiple species, such as CAMPR3 (27), APD3 (28), AMPer (29), DAMPD (15), ADAM (20) and LAMP (30). A little number of databases dedicated to the function-specific AMPs, e.g. Antiviral Peptides (AVPdb (31)), Defensins Knowledgebase (32), synthetic peptides (SAPD (33) and DBAASP (34)), and recombinantly-produced AMPs (RAPD (16)). An increasing development of AMP databases has promoted many approaches dedicated to in silico prediction of AMPs based on amino acid composition (35). Recently, the advent of high-throughput technologies has led molecular biology into a data surge in both growth and scope (36). For instance, the next-generation sequencing (NGS) technology has been applied to generate large-scale DNA/RNA reads from foods (37), water (38), soil, air and specimen, for identifying microbiota and their functions based on metagenomics and metatranscriptomics, respectively. Additionally, mass spectrometry (MS) was also widely applied in proteomics studies for generating thousands of peptides in one experiment. Rapidly advancing technologies have offered us the opportunities to examine the genome, transcriptome, and proteome in comprehensive ways. Thus, we were motivated to design a database-assisted system (dbAMP: http://csb.cse.yzu.edu.tw/dbAMP) for exploring AMPs with functional activities and physicochemical properties on transcriptome and proteome data.

## MATERIALS AND METHODS

**Figure 1.**
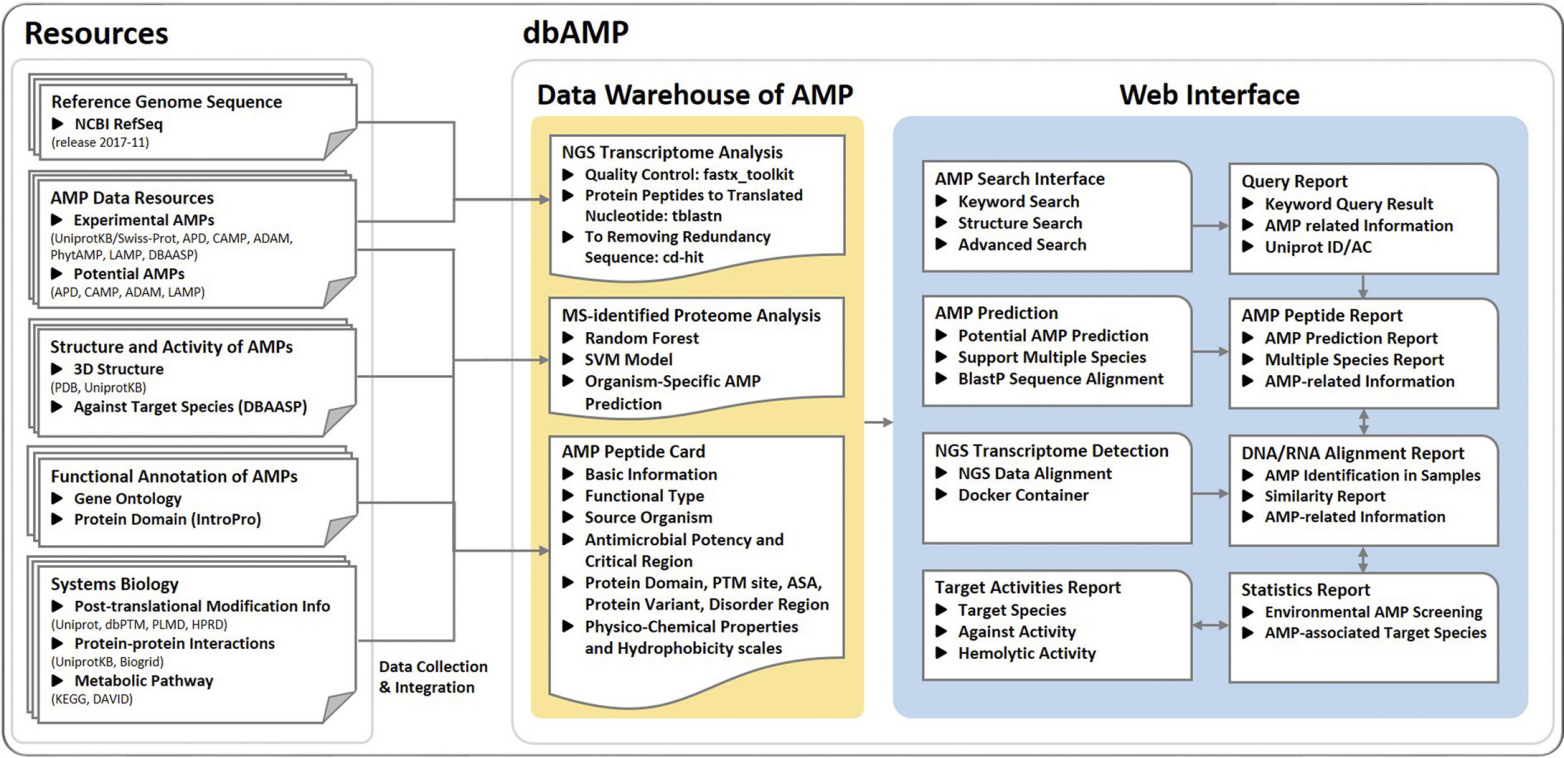
Schematic flowchart of dbAMP construction including data integration, functional analyses and web interface development.

The dbAMP is an open-access and manually curated database harboring diverse annotations of AMPs including sequence information, antimicrobial activities, post-translational modifications (PTMs), structural visualization, antimicrobial potency, target species with minimum inhibitory concentration (MIC), physicochemical properties, AMP–protein interactions, as well as the supporting references. In addition to the functional and physicochemical annotations, dbAMP provides an effective AMP prediction on proteome data from different species and a large-scale AMP detection on transcriptome data from NGS technologies. The flowchart of dbAMP construction is presented in Figure 1, including data integration and curation, functional and physicochemical analyses, characterization and identification of AMPs on different species, detection of AMPs on transcriptome data, and the development of dbAMP web interface. Moreover, a couple of external databases related to AMP functions are also integrated into the proposed resource.

### Integration of antimicrobial peptides from public domain and literature

Both experimentally validated and putative AMPs were retrieved from protein database of NCBI (39), UniProt (40), Protein Data Bank (41) and public AMP databases such as APD3 (28), CAMPR3 (27), ADAM (20), PhytAMP (22), AMPer (29), AntiBP2 (42), BACTIBASE (24) and LAMP (30). After the removal of redundant sequences by mapping all collected AMPs to UniProt protein entries, a total of 12,389 unique AMPs were integrated into the proposed resource which contains 4271 experimentally verified AMPs along with their functional activities obtained from 2048 organisms. In addition to databases integration, digging knowledge concerning AMPs in the related articles can enable a full understanding of the functional activities of AMPs and their targets. However, the surge in scope and scale of PubMed literature database has conducted a formidable challenge in manually curating AMPs from research articles. Thus, we designed a pipelined text extraction system for retrieving AMP-related articles by querying appropriate keywords such as ‘antimicrobial’, ‘antibacterial’, ‘anti-gram positive bacterial’, ‘anti-gram negative bacterial’, ‘antifungal’, ‘antiviral’, ‘antiparasitic’, ‘antibiofilm’, ‘antimalarial’, ‘antiprotozoal’, ‘antiyeast’, ‘anticancer’, ‘antitumor’, ‘wound healing’, ‘spermicidal’, ‘insecticidal’ and ‘surface immobilized’ against searchable fields ‘Title’, ‘Abstract’ and ‘Keywords’ of PubMed literature database. After obtaining the potentially AMP-related articles, an approach of name entity recognition (43) was adopted to detect name entities, which are summarized from validated AMPs existed in dbAMP, in the text and label them with appropriate tags. Then, a natural language processing algorithm (31) was employed to extract relations among those entities for obtaining functional activities and target species of AMPs. After that, the articles labeled with specified name entities and functional contexts were further manually curated. Up to July 2018, a total of 1924 references were extracted and regarded as the evidence for supporting dbAMP data entries.

### Comprehensive analyses for functional and physicochemical properties

An increasing interest in the functional and physicochemical investigation of AMPs motivated the mapping of all AMPs onto protein entries of UniProt and Protein Data Bank (PDB) based on sequence identity, which enables users to examine amino acid composition, post-translational modifications (PTMs), functional domains, solvent-accessible surface area, secondary structure, AMP–protein interactions, hydrophobicity, as well as the composition of positively and negatively charged residues. As for functional domains, the InterPro (44) is an integrated database for annotating ‘signatures’ like protein families, domains, and functional sites on proteins. It has been reported that a protein-interacting domain usually recognizes a short peptide motif of target protein but does not bind stably until the peptide has an appropriate PTM; this can create binding sites for specific protein-interaction domains that work together for cellular function (45). The information of PTMs on AMPs was obtained from dbPTM (46), which has accumulated most comprehensive data for validated substrate sites of various PTM types.

An increasing number of studies suggested that AMPs can play multiple roles not only in the interaction with membrane lipids and proteins but also in the intracellular targeting mechanisms, which include nucleic acids and protein biosynthesis, protein-folding, protease, cell division, cell wall biosynthesis and lipopolysaccharide inhibition (47,48). Thus, one of the aims in this resource is integrating the information of physical protein-protein interactions (PPIs) to explore the potentially intracellular targeting proteins of AMPs. For this purpose, the information of validated physical interactions was obtained from over ten PPI databases (as listed in Supplementary Table S1). Additionally, with an attempt to understand the potential target species and activity of AMPs, the BLAST program was used to query dbAMP peptides against the DBAASP database (34), with 100% identity and e-value <1e10^–5^ as the threshold. In DBAASP data content, the information of AMP activity and cytotoxicity were extracted from published papers where the minimal inhibitory concentration (MIC, μM), based on microdilution assays, was reported for Escherichia coli, Staphylococcus aureus, or Pseudomonas aeruginosa. In order to increase the use of dbAMP for clinical treatment, cytotoxicity data with the measurement of hemolytic activity (HC) value for each AMP has been stored in our database. The HC50 value refers to peptide concentration, which is annotated as micromolar and is required for 50% haemolysis of red blood cells (RBCs). Each peptide has been annotated with different hemolytic potencies on different RBCs, if available.

### Detection of critical region of antimicrobial potency

It has been reported that multicellular eukaryotes can secrete AMP-Releasing Proteins (AMP-RPs), which release active peptides after a partial proteolytic processing conducted by bacterial or host proteases (49–51). Computationally identifying the presences of putative antimicrobial regions inside a larger protein (AMP precursor) could be a useful scheme for the exploration of new cryptic AMPs naturally encoded from host genome (52). Hence, we have adopted an effective sliding window analysis to search for critical region of antimicrobial potency contained into the primary structure of larger proteins and precursors. This work focused on the identification of a critical antimicrobial region endowed with a significant antimicrobial potency for discovering cryptic AMP from host proteins. Pane *et al.* have demonstrated that the antimicrobial potency is highly correlated to the formula: *C^m^H^n^L* where *C* stands for the net charge of a peptide, *H* represents a measure of its hydrophobicity and *L* is peptide length (52). Coefficients m and n are conducted by accounting for the influence of bacterial strain features and environmental conditions on the interaction between the AMP and a cellular membrane. The Relative AntiMicrobial Score (RAMS) for a given peptide can be determined by:

(1) \begin{equation*}{\rm{RAMS}} = {C^m}\;{H^n}/MaxScore\end{equation*}

where *C* is the sum of net charge and *H* is the sum of hydrophobicity scores in a sliding window along the peptide. *MaxScore* is the maximal value of ${C^m}{H^n}$ score which can be obtained from a given peptide at optimal values of *m* and *n*, evaluated in a suited range. According to the evaluation of correlation coefficient between RAMS values and experimental antimicrobial potency values, the optimal values for *m* and *n* are 0.9 and 1.1, respectively. However, most AMPs are of diversity in sequence length. In order to formalize various antimicrobial scores owing to different peptide lengths, we provided a flexible scheme to analyze all possible peptide lengths ranging from 12 to 40 amino acid residues in a protein sequence with a sliding window analysis (52,53). The absolute RAMS (ARAMS) can thus be calculated for a given peptide length *L*, which is defined as (54):

(2) \begin{equation*}{\rm{Absolute\, RAMS}}\ \left( {{\rm{ARAMS}}} \right) = {\rm{RAMS}} \times {\rm{L}}\end{equation*}

The antimicrobial scoring function was carried out on all AMPs through the sliding window analysis. To present a clear investigation of antimicrobial potency on AMPs, an isometric plot was created to show the ARAMS values based on different window lengths, ranging from 12 to 40 amino acid residues, for each AMP sequence using parameters optimized for strain *S. aureus* C623. As presented in Supplementary Figure S1, the x axis stands for different values of window size and y axis labels the amino acid sequence of the peptide. A sliding window having ARAMS value higher than 3.0 in response to having MIC value lower than 200 μM is highlighted with blue color.

### Investigation and identification of AMPs on different source species

Due to no existing resources dedicated to the prediction of AMPs on different source organisms, in addition to providing a user-friendly interface for browsing the collected AMPs in dbAMP, all the experimentally verified AMPs were utilized to generate AMP-prediction models on different host species, including bacteria, humans, amphibian, fish, plants, insects, and mammals, based on random forest (RF) algorithm. As depicted in Supplementary Figure S2, the positive (AMPs) and negative (non-AMPs) training datasets, used for the construction of predictive models, were constituted from dbAMP and UniProt entries, respectively. After the removal of homologous training sequences by using CD-HIT program (55) with 40% identity, the resulting numbers of positive training sequences for human, amphibia, fish, insect, plant, bacterial, and mammal are 232, 926, 118, 274, 454, 431 and 559, respectively. Because only 35 peptides are annotated with experimentally-verified no antimicrobial activity in UniProt, we followed the data preparation procedure conducted in other studies (56–59) to generate our negative dataset. The protein sequences, containing sequence length between 10 and 100 and annotated without the information of membrane, toxic, secretory, defensin, antimicrobial, antibiotic, anticancer, antiviral and antifungal, were obtained from UniProt and regarded as the non-AMPs for the seven studied species. Consistent with the processing criteria used in positive training dataset, the CD-HIT program was adopted again to remove homologous sequences among non-AMP sequences, based on sequence identity of 40%. Additionally, 20% of positive and negative training sequences were randomly selected to compose the positive and negative testing datasets, respectively. A summary of numbers of training dataset (80%) and testing dataset (20%) is given in Supplementary Table S2.

Both amino acid composition (60,61) and physicochemical properties (62) were considered as the attributes for characterization and identification of AMPs on seven species. In this analysis, the random forest (RF), a sort of ensemble model that involves the aggregation of multiple decision tree classifiers, was employed to generate predictive models. Based on the integration of multiple decision trees within a RF model, each tree was generated from a subset of *k* attributes randomly selecting from training dataset with a total of m attributes, where *k* is less than *m*. In this way, we can obtain multiple decision-making results. Typically, the majority voting method is adopted to integrate the results to make a final decision, based on the class label with the most votes. The performance of a RF decision-making system is associated with the dimension of random vector, which is the number of attributes (*k*) used in each decision tree. The value of k is typically defined as

(3) \begin{equation*}{{k}} = {{\log}}{{}_{{\bf 2}}}\;{{m}} + {{1}}\end{equation*}

where *m* is the total number of attributes in training dataset (63). In this study, a package of random forest, which has been integrated into Weka toolkit (64), was utilized to construct RF classifiers based on various attribute sets. In the generation of RF models, the *k*-fold cross-validation was employed to evaluate their predictive performances. After the evaluation of *k*-fold cross-validation, the RF model reaching a best predictive performance was further evaluated by the testing dataset, which is totally independent to the training dataset.

### Large-scale detection of AMPs on transcriptome data

**Figure 2.**
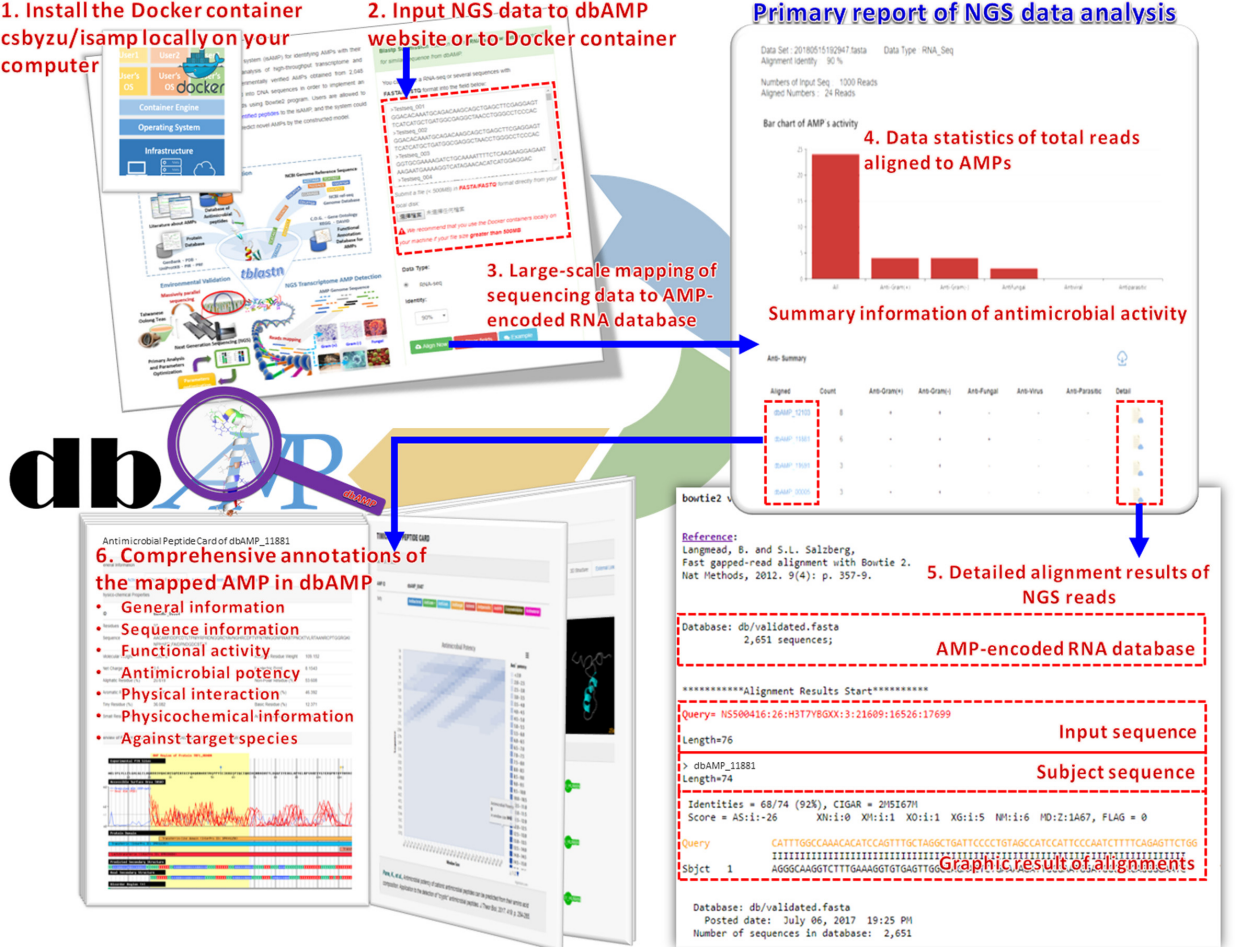
Flowchart of using the developed Docker container ‘*csbyzu/isamp’* to detect AMPs in NGS data.

An increasing interest of identifying natural AMPs enables the development of new antibiotics. The emerging NGS was widely utilized to obtain large-scale DNA or RNA sequencing datasets from various samples such as food, water, soil, air and specimen, for conducting genomic or transcriptomic analyses (65). The high-throughput RNA sequencing technology offered us an opportunity to discover cryptic AMPs in the transcriptome data obtained from environments or experiments. Thus, we were motivated to design a database-assisted system for identifying AMPs with their functional types based on the metatranscriptomic analysis of various transcriptome data. For efficiently searching out RNA reads that potentially encode for AMPs, all the amino acid sequences of experimentally verified AMPs were transformed back to RNA sequences for constructing an AMP-encoded RNA database by using BLAST program with 100% identity, 80% sequence coverage, and e-value <1e10^–5^ as the threshold. This resulted in a total of 2651 AMP-encoded RNA sequences with functional activities retrieved from their original annotations of AMPs. Then, the Bowtie2 program (66) was integrated to implement a downloadable pipeline for discovering AMPs from NGS sequencing data, based on a Docker virtualization software that can package applications and their dependencies in a virtual container running on Linux server (67,68). The developed container *csbyzu/ismap* has been uploaded onto the Docker open source, which enables flexibility and portability on where the package can run. As presented in Figure 2, users can submit a large-scale data of NGS reads or MS/MS-identified peptides to the Docker container *csbyzu/isamp* locally on your computer, and the package could identify known AMPs with their functional activities and predict novel AMPs by the constructed RF models. The analyzed results, including data statistics of total reads aligned to AMPs, summary table of functional activities, detailed alignment results of NGS reads, and comprehensive annotations of the mapped AMP in dbAMP, can be displayed by the web interface which has been integrated into the Docker container.

## DATABASE CONTENT AND UTILITY

### Data statistics of antimicrobial peptides in dbAMP

The dbAMP was created as a useful resource for accumulating natural and synthetic AMPs from scientific literature and published AMP databases. One of the aims of dbAMP is to provide most comprehensive information of AMPs as well as their antimicrobial activities and physicochemical characteristics. Currently in dbAMP there are 12 389 unique entries, including 4271 experimentally verified AMPs and 8118 putative AMPs along with their functional activities, supported by 1924 research articles. Each unique AMP sequence was assigned an identification number (i.e. dbAMP ID) beginning with the prefix ‘dbAMP’. The basic information related to AMPs, including functional activities, physicochemical properties, taxonomy of the source organism, target species, PDB structures, and crosslinks to external databases, can be accessed via dbAMP ID. Table 1 summarizes the number of AMPs according to their functional activities in dbAMP as well as in other databases. Currently dbAMP has a repository of over 20 types of functional activities on AMPs. It is worth mentioning that 114 anti-MRSA peptides (69), a newly annotated type of antimicrobial activity, has been integrated into dbAMP.

**Table 1.** Comparison of data statistics between dbAMP and other AMP databases based on functional activities of AMPs

Functional activity	dbAMP	DBAASP	APD	CAMP	ADAM	PhytAMP	LAMP
**Antibacterial**	3006	2232	1536	2914	2350	52	-
**Anti-Gram_positive**	2726	2196	2124	1901	2080	-	-
**Anti-Gram_negative**	2323	2142	1722	1743	1913	-	-
**Anti-fungal**	1623	1433	1187	1150	1181	85	90
**Anti-viral**	300	85	186	117	181	15	78
**Anti-parasitic**	123	83	111	35	95	-	4
**Anti-HIV**	109	-	109	-	-	-	-
**Wound Healing**	19	-	18	-	-	-	-
**Chemotactic**	59	-	59	-	-	-	-
**Cancer Cells**	227	219	210	22	-	-	-
**Anti-biofilm**	40	-	31	-	-	-	-
**Antimalarial**	26	-	24	-	-	-	-
**Antioxidant**	22	-	22	-	-	-	-
**Antiprotozoal**	6	-	4	-	-	-	-
**Spermicidal**	13	-	13	-	-	-	-
**Insecticidal**	35	32	33	-	-	4	-
**Antimicrobial**	4816	-	494	4812	-	-	2840
**Enzyme Inhibitor**	26	-	26	-	-	-	-
**Anti-tumour**	9	-	4	8	-	-	-
**Mammalian Cells**	308	-	308	-	-	-	-
**Surface Immobilized**	19	-	19	-	-	-	-
**Anti-oncogenic**	1	-	-	-	-	-	-
**Sodium Channel Blocker**	2	-	-	-	-	2	-
**Anti-yeast**	4	-	-	-	-	4	-
**Anti-inflammatory**	2	-	-	-	-	-	-
**Anti-MRSA**	114	-	-	-	-	-	-

### Sequential properties of AMPs

**Figure 3.**
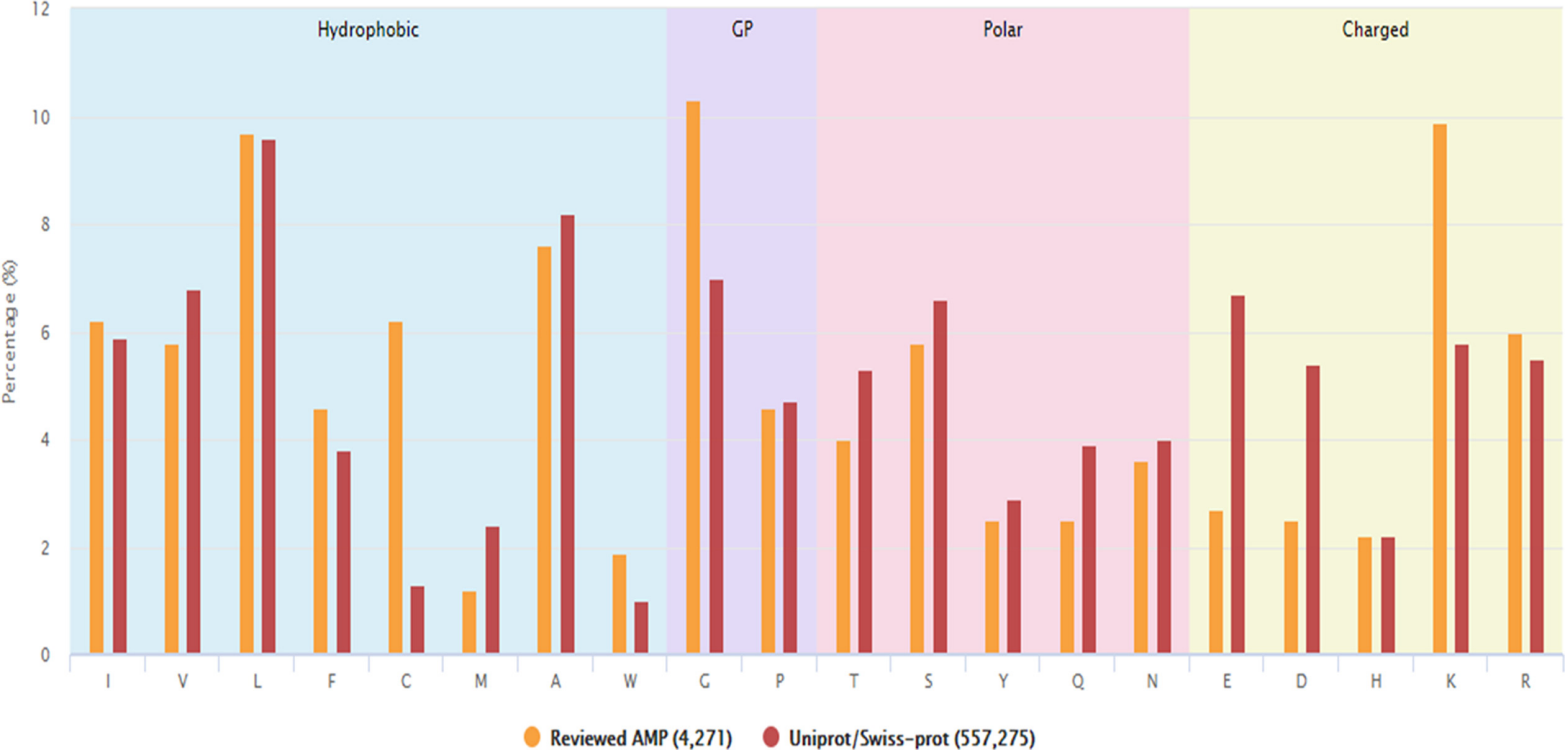
Comparison of amino acid composition between validated AMPs in dbAMP and a background set of reviewed proteins obtained from UniProt.

With the infrastructure of integrating comprehensively validated data into dbAMP, we can conduct a full investigation of sequence-based features, such as sequence length and amino acid composition (AAC), on AMPs according to different source species. The distribution of AMP source organisms is illustrated in Supplementary Figure S3. Nearly 90% of the experimentally verified AMPs contain 80 amino acid residues or less with an average peptide length of 46. A detailed distribution of peptide length of validated AMPs has also been given in Supplementary Figure S4. Existing methods have demonstrated that the composition of amino acids is a potent characteristic for AMP identification (59,70); therefore, we compared amino acid composition profile between 4271 validated AMPs and a background set of reviewed proteins obtained from UniProt. Figure 3 shows the leucine (L), glycine (G) and lysine (K) are the top three abundant amino acids. The frequencies of Cysteine (C), G and K residues are significantly higher in AMPs. The cysteine-rich antimicrobial peptides are containing a couple of disulfide bonds for stabilizing its tertiary structures in plants and invertebrates (71–73). The glycine-rich AMPs, such as acanthoscurrin (74), hyastatin (75) and armadillidin H (76), induce a higher frequency of G residue in AMPs. The enrichment of K residue contributes positive net charge onto hydrophilic side of amphipathic AMPs for attaching to the membrane surface of microbes (77). A partial reason constitutes the positive net charge in AMPs is owing to the lack of aspartic (D) and glutamic (E) acids. In addition, Supplementary Figure S5 presents the amino acid composition of AMPs based on different species.

### Physicochemical properties of AMPs

**Figure 4.**
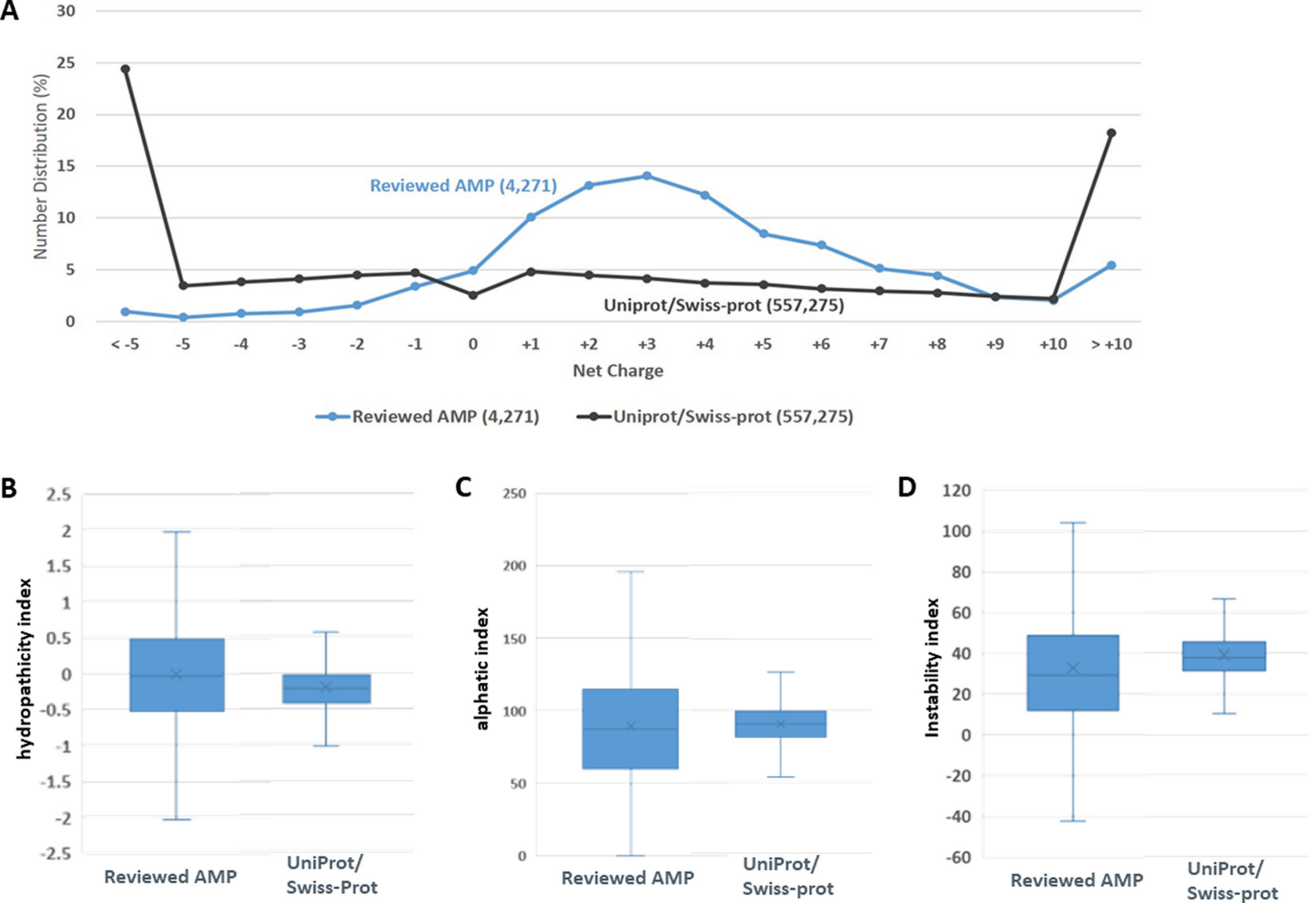
Physicochemical properties of validated AMPs. (**A**) Comparison of net charge distribution between dbAMP validated peptides and UniProt reviewed proteins. Comparisons of box plots of (**B**) hydropathicity, (**C**) alphatic and (**D**) instability indices between dbAMP validated peptides and UniProt reviewed proteins.

Antimicrobial peptides occur naturally as important innate immunity agents in a wide range of living organisms, and they are characterized by their positive net charge, modest length, and good solubility in water (78). As presented in Figure 4A, the comparison of net charge distribution between dbAMP and UniProt entries has revealed that a majority of AMPs had net charge values between +2 and +4 and less than 5% AMPs had a negative net charge value, which is consistent with a previous discovery (79). The average value of net charge of AMPs is around 3. The information of AMP tertiary structures was retrieved from PubMed or UniProt with a database ID crosslinking to PDB. Moreover, the EMBOSS pepinfo (80) has been integrated to obtain general information such as molecular weight, isoelectric point, charge, hydrophobicity values, as well as the composition of positively and negatively charged residues. Comparison of box plots of hydropathicity indices between dbAMP validated AMPs and UniProt reviewed proteins is depicted in Figure 4B. The information of hydrophobicity and net charge are considered as the crucial attributes for AMPs (81). Additionally, Figure 4C and D provides the comparisons of aliphatic and instability indices, respectively, for validated AMPs and UniProt reviewed proteins. Overall, these comparisons indicate that the validated AMPs contain more diverse values of hydropathicity, aliphatic and instability indices when comparing to UniProt reviewed proteins. Supplementary Figure S6 further shows that the net charge distributions of validated AMPs are noticeably diverse among different source species. In order to identify useful features for classifying between AMPs and non-AMPs on multiple species, the compositions of hydrophobic residues on different regions of AMPs, ranging from N-terminal end to C-terminal end, are provided in Supplementary Figure S7. Due to the variously physicochemical characteristics on different source species, this work assigned an antimicrobial score to AMPs based on their net charge, hydrophobicity and length factors. This physicochemical investigation enables us to localize the position of cryptic peptides, for yielding an accurate map of the molecular determinants of their antimicrobial activity.

### Functional analysis of AMPs

An increasing number of studies have suggested that AMPs can play the roles in the intracellular targeting mechanisms (47,48). For instance, the human antimicrobial peptide elafin domain can interact with other intracellular proteins for specifically participating into the apoptosis induction mechanism in melanoma cells (82,83). Targets of AMPs may be either bacterial membranes or diverse intracellular molecules; however, some peptides can operate through complex mechanisms that involve multiple targets (84). Hence, the dbAMP has integrated the information of functional domains and PPIs to explore the interactions between AMPs and potentially targeting proteins. Currently the dbAMP has accumulated 6,535 proteins that had the experimental evidence of physically interacting with AMPs, which provides a starting point for discovering potential targets of AMPs. Moreover, in order to explore the functions of potential AMP-targeting proteins, functional enrichment analysis (FEA) was performed by using the DAVID functional annotation tool (85). Next, gene ontology (GO) analysis was conducted to examine the dominant functions in biological processes, cellular components, and molecular functions. The distribution of top 20 GO terms for 6535 AMP-interacting proteins is displayed in Supplementary Figure S8. This functional analysis revealed that the potential AMP-targeting protein tends to be affected in terms of nucleotide metabolism and signaling pathways with response to regulations of signal transduction, cell adhesion, viral infection and apoptotic process.

### Computational identification of AMPs on proteome data

It has been fully investigated that the dominant net charge and amino acids composition differ in various species. Machine learning (ML) method has been utilized to generate predictive models. Based on the evaluation of 5-fold cross-validation, four ML algorithms have been adopted to make the comparison of predictive performance in different species. Supplementary Table S3 shows that the proposed RF models could reach highest prediction accuracy on proteome data of different species. Accuracies for all the seven organisms are observed as higher than 93% with balanced sensitivities and specificities. This investigation has indicated that the RF model trained with the examined attributes could provide accurate predictions against multiple species. Moreover, Supplementary Table S4 also demonstrates that the proposed RF models could provide promising accuracies in different species, based on the independent testing dataset. To our knowledge, a variety of computational methods have been proposed for the prediction of antimicrobial peptides. However, there exists no online resource dedicated to characterizing and identifying AMPs on different source species, such as bacteria, humans, amphibian, fish, plants, insects and mammals. The dbAMP allows users to input a single sequence or multiple sequences with FASTA format or to upload a text file to perform the AMP prediction by specifying a species or life domain on the prediction page of website.

### Detection of AMPs on NGS transcriptome data

Many methods have been developed to perform metagenomic and metatranscriptomic data analysis for NGS raw data. For instance, the UPARSE pipeline constructs a set of operational taxonomic unit from NGS amplicon reads used to understand the microbial community structure (86). The MG-RAST server is a SEED-based algorithm that characterizes the taxonomic composition, functional potential and diversity of the microbial assemblages (87). In contrast, we designed a database-assisted system specialized in identifying AMPs and their functional activities based on metatranscriptomic analysis of high-throughput transcriptome data. The dbAMP provides an intuitive graphical user interface (GUI) to execute a Docker container csbyzu/isamp on local machine. Metagenomics and metatranscriptomics analyses of diverse microscopic organisms in natural environments, including human body, have revolutionized the understanding of the relationship between microbes and their hosts. To showcase the new scheme of AMPs discovery, the RNA sequencing samples of Taiwanese oolong teas (Dayuling, Alishan, Jinxuan and Oriental Beauty teas), obtained from NCBI SRA with accession number SRP113601 (88), were subjected to the quality control and sequence alignment against dbAMP entries. As shown in Supplementary Table S5, among the reads mapped to plants, totally 1 077 775 (9.1%), 1 305 016 (6.8%), 1 152 178 (8.4%) and 366 454 (7.4%) RNA reads could be mapped to AMPs with sequence identity of 100% in Dayuling, Alishan, Jinxuan and Oriental Beauty teas, respectively. On the other hand, among the reads mapped to bacterial, a total of 8194 (6.5%), 26 220 (6.2%), 5753 (6.1%) and 106 683 (7.7%) RNA reads could be mapped to AMPs with sequence identity of 100% in Dayuling, Alishan, Jinxuan and Oriental Beauty teas, respectively. Furthermore, Supplementary Figures S9A presents the distribution of anti-Gram-positive and anti-Gram-negative AMPs of plants in four oolong teas. In addition, Supplementary Figures S9B also shows the distribution of anti-Gram-positive and anti-Gram-negative AMPs of bacterial in four oolong teas. As presented in Supplementary Figure S9C, the composition of anti-Gram-positive AMPs in Jinxuan and Oriental Beauty teas is more abundant than that in Dayuling and Alishan teas. Meanwhile, the composition of Gram-positive bacterial in Jinxuan and Oriental Beauty teas is less than that in Dayuling and Alishan teas.

### Web interface of dbAMP

**Figure 5.**
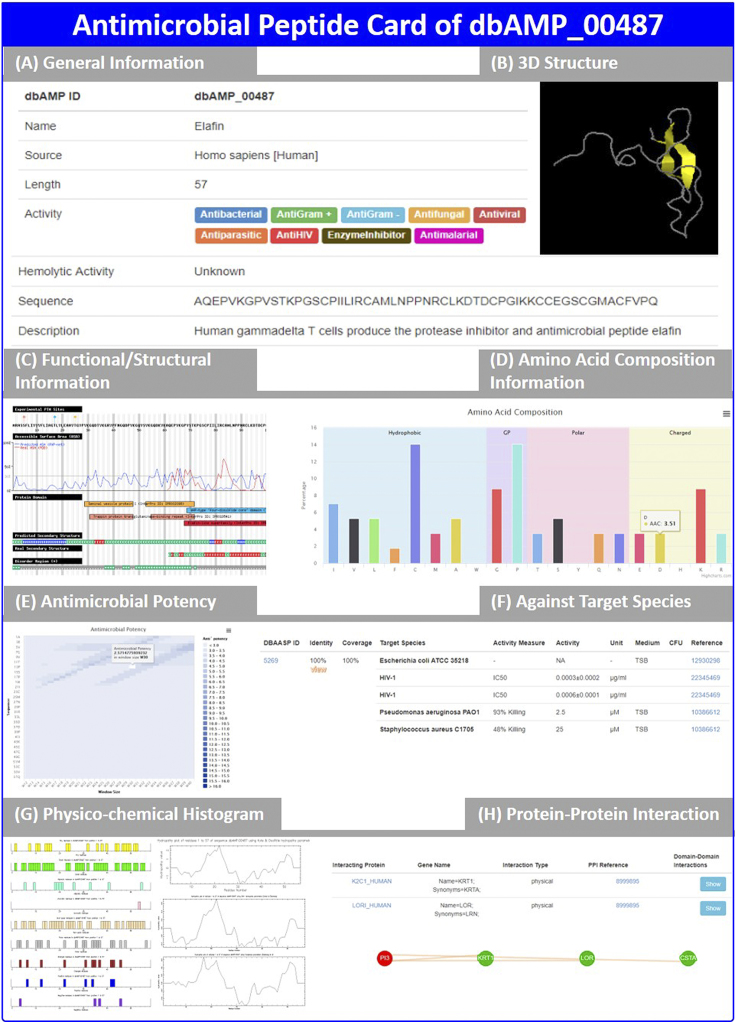
Web interface of displaying comprehensive annotations for human antimicrobial peptide elafin. The comprehensively structural and functional analyses include (**A**) general information, (**B**) visualization of AMP tertiary structure, (**C**) solvent accessibility and functional domains, (**D**) amino acid composition, (**E**) antimicrobial potency, (**F**) antimicrobial target species, (**G**) histogram of hydrophobicity and composition of positively and negatively charged residues, as well as (**H**) AMP–protein interactions.

To enable the comprehensive analyses of AMPs, the dbAMP has provided users the web interface with enhanced designs. The dbAMP was built on the Centos (CentOS release 6.9 Final) Linux operating system using the freeware Apache (Apache/2.2.15) web server, PHP programming language, and MySQL database system (5.5.56 MySQL Community Server). All the collected AMPs have been manually curated and systematically stored on the database management system of MySQL. In order to visualize AMP tertiary structures obtained from both the PDB and molecular dynamics (MD) simulations, the Jmol program (89) has been integrated into the PHP web program. The web interface also constructs a couple of links connecting to external resources, including UniProtKB, PubMed, PDB, Pfam (90), as well as other AMP databases, for providing additionally functional information. Users are allowed to browse all the AMPs and submit RNA sequencing reads or MS/MS-identified peptides to the dbAMP, and the system could identify known AMPs with their functional activities and discover novel AMPs by the predictive models. Figure 5 showcases the web interface providing comprehensively functional and physicochemical analyses.

## CONCLUSION

In recent years, many studies have been conducted to dig out new drugs for multidrug-resistant bacteria. The results of these studies have concluded that antimicrobial peptides have a high potential to be considered as an alternative drug for conventional antibiotics and become a computational model for the development of new antimicrobial drugs that can solve the problem with regarding to an increasing number of multidrug resistances of pathogenic microorganisms (91). However, it is labor-intensive and time-consuming to design experimental methods to discover natural AMPs. We are thus inspired to design a database-assisted platform (dbAMP) for providing comprehensively functional and physicochemical analyses for AMPs based on the large-scale transcriptome and proteome data. Table 2 shows the comparison of system functionalities between dbAMP and other AMP databases. Significant improvements available in dbAMP comprise the information of AMP–protein interactions, antimicrobial potency analysis for ‘cryptic’ region detection, annotations of AMP against target species and AMP detection on transcriptome and proteome datasets. To our knowledge, the dbAMP is the first resource for providing a downloadable package to discover known and novel AMPs on high-throughput omics data. Additionally, user-friendly visualization interfaces have been designed to facilitate peptide searching, browsing and sequence alignment against dbAMP entries. All in all, the dbAMP can promote functional analyses of antimicrobial peptides and become a valuable resource for the discovery of new antimicrobial drugs.

**Table 2.** Comparison of system functionality between dbAMP and other AMP databases

Database	${\rm{CAM}}{{\rm{P}}_{{\rm{R}}3}}$	APD3	ADAM	LAMP	AMPer	AntiBP2	BACTIBASE	PhytAMP	dbAMP
**PubMed ID**	26467475	26602694	26000295	23825543	17341497	20122190	20105292	18836196	-
**Number of AMP sequences**	10 247	2889	7007	5548	1298	999	228	273	12 389
**Number of organisms**	773	1396	794	Multiple	Eukaryotes	Multiple	Bacteriocins	Plant	2048
**Number of AMP tertiary structures**	757	388	759	189	N/A	N/A	72	39	1169
**AMP prediction model**	Random forest	-	SVM/HMM	-	HMM	SVM	-	HMM	Random forest
**AMP detection on multiple species**	N/A	-	N/A	-	N/A	N/A	-	N/A	Seven life domains
**Number of AMP targets**	-	-	-	-	-	-	-	-	2172
**Number of AMP-interacting proteins**	-	-	-	-	-	-	-	-	6338
**Antimicrobial potency Analysis**	-	-	-	-	-	-	-	-	Antimicrobial potency of cationic AMPs can be determined from their amino acid composition
**Detection of cryptic region in AMPs**	-	-	-	-	-	-	-	-	Determining the cryptic region by referring to net charge and hydrophobicity
**Against target species**									Providing the activity of AMPs against target species based on sequence analysis
**NGS data analysis**	-	-	-	-	-	-	-	-	Using the developed Docker container to explore AMPs for transcriptome data

## DATA AVAILABILITY

The data content in dbAMP will be maintained and updated quarterly by continuously surveying the public resources and research articles. The database-assistant system is now freely accessed online at http://csb.cse.yzu.edu.tw/dbAMP/. All of the experimentally verified AMPs as well as the putative AMP dataset could be downloaded in the text format. Additionally, the Supplementary Figures S1–S9 and Tables S1–S5 are available at NAR online.

## Supplementary Material

Supplementary Data
