# Supplementary material for: dbAMP: an integrated resource for exploring antimicrobial peptides with functional activities and physicochemical properties on transcriptome and proteome data
Source: Nucleic Acids Res. 2018 Oct 31;47(Database issue):D285–97. doi: 10.1093/nar/gky1030 (PMC6323920; doi:10.1093/nar/gky1030)
Supplement: Supplementary Data [file gky1030_supplemental_files.docx]

**SUPPLEMENTARY MATERIALS**

**Figure S1. An isometric plot displaying the ARAMS values based on different window lengths.**

**Figure S2. Flowchart of performing computational prediction of AMPs on different species.**

**Figure S3. The distribution of AMP source organisms in dbAMP.**

**Figure S4. Detailed distribution of peptide length of validated AMPs in dbAMP.**

**Figure S5. Investigation of amino acid composition of AMPs based on different species.**

**Figure S6. Investigation of net charge distribution of AMPs based on different species.**

**Figure S7. Compositions of hydrophobic residues on different regions of AMPs based on their source species.**

**Figure S8. Distribution of top 20 GO terms for AMP-interacting proteins.**

**Figure S9. Distribution of AMPs in four oolong teas. (A) The distribution of anti-gram-positive and anti-gram-negative AMPs in four oolong teas. (B) The distribution of gram-positive and gram-negative bacterial in four oolong teas.**

**Table S1. Data statistics of integrated protein-protein interaction databases.**

**Table S2: Data statistics of training and testing datasets used for constructing and evaluating the predictive models, respectively, on seven species.**

**Table S3. Comparison of five-fold cross-validation results of four machine learning methods in different organisms.**

**Table S4. Cross-validation and independet testing results of the generated RF models against multiple species.**

**Table S5. Data statistics of RNA reads mapped to AMPs.**


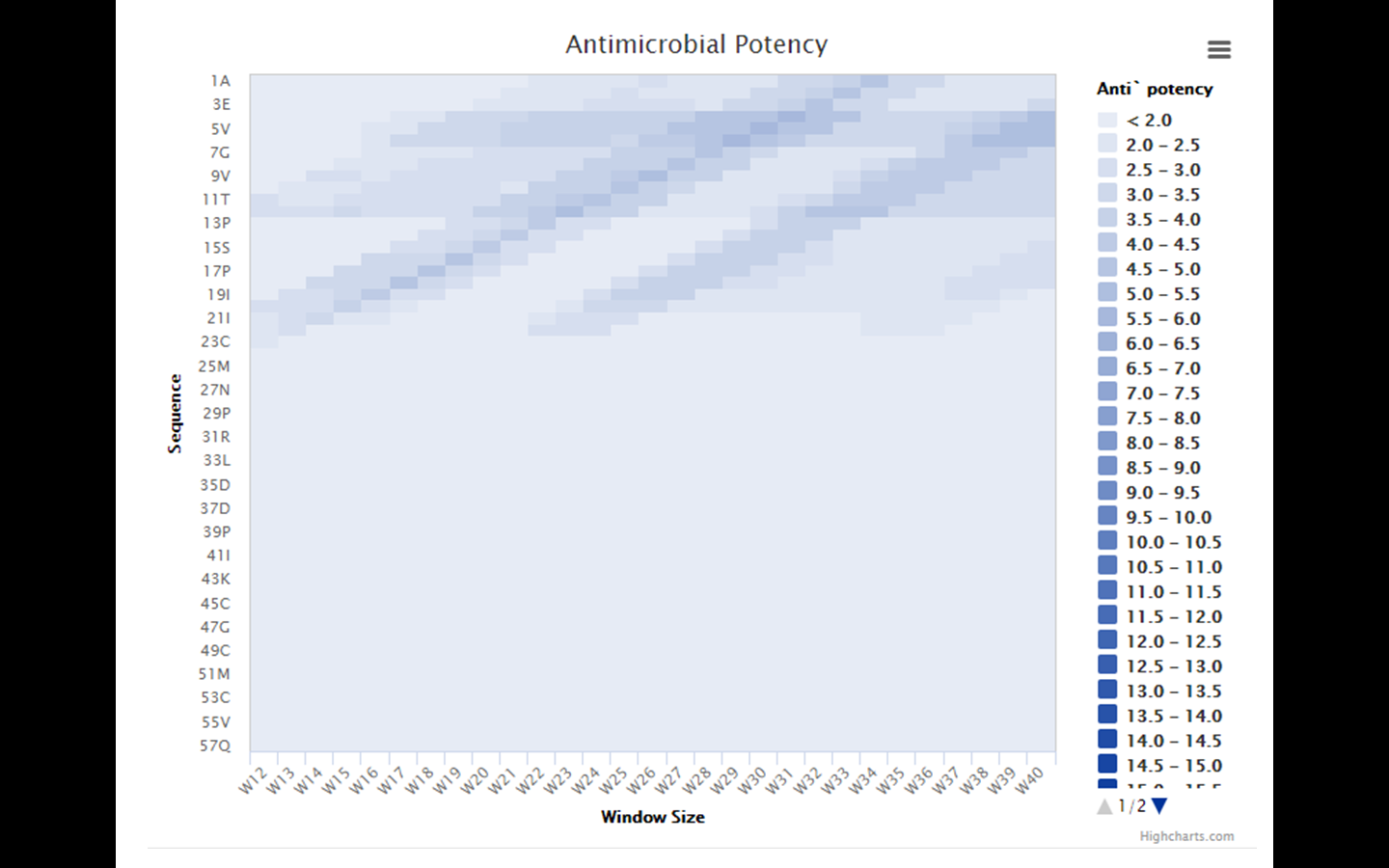


**Figure S1. An isometric plot displaying the ARAMS values based on different window lengths.** The x axis stands for different values of window size and y axis labels the amino acid sequence of the peptide. The sliding window having ARAMS value higher than 3.0 in response to MIC value lower than 200 μM is highlighted with blue color. This case shows that the peptide contains a higher antimicrobial potency in N-terminal region.


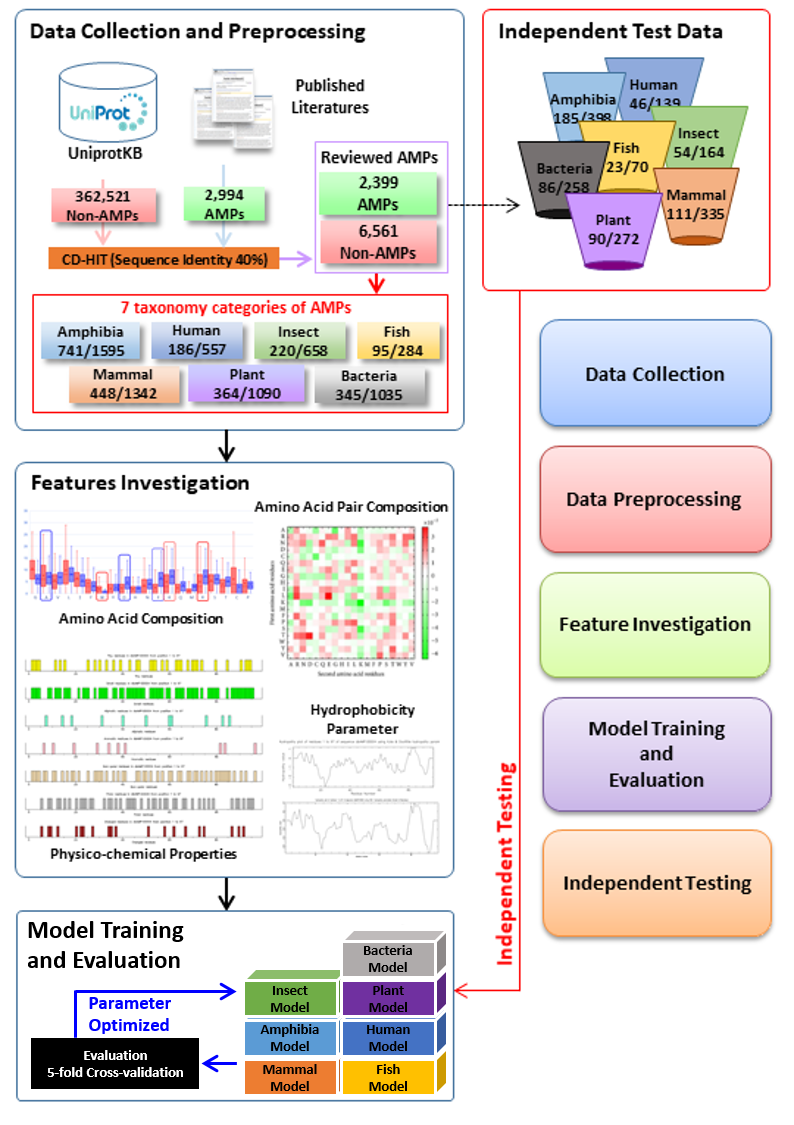


**Figure S2. Flowchart of performing computational prediction of AMPs on different species.** In the generation of RF models, the *k*-fold cross-validation was employed to evaluate their predictive performances. In the implementation of *k*-fold cross-validation, all the training data, including positive and negative sequences, were randomly clustered into k equal-sized subgroups. After having k subgroups, k-1 of them shall be regarded as the training sample and the remaining one subgroup was considered as the validation sample. In a round of k-fold cross-validation, each of k subgroups should be considered as the validation sample once in turn. Sensitivity (*Sn*), specificity (*Sp*), accuracy (*Acc*), and Matthews correlation coefficient (MCC) have been used as the metrics to determine the performance of the generated models. The four metrics are defined as: $=\frac{TP}{TP+FN}$ , $Sp=\frac{TN}{TN+FP}$ , $Acc=\frac{TP+TN}{TP+FP+TN+FN}$ , and $MCC=\frac{\left( TP\times TN \right)-(FN\times FP)}{\sqrt{(TP+FN)\times(TN+FP)\times(TP+FP)\times(TN+FN)}}$ , where TP, FN, TN, and FP denote the instances of true positive, false negative, true negative, and false positives, respectively. Due to the unbalanced positive and negative training datasets in this work, we have decided to choose MCC value as a major benchmark for achieving a relatively balanced sensitivity and specificity. After the evaluation of *k*-fold cross-validation, the RF model reaching a best predictive performance was further evaluated by the testing dataset, which is totally independent to the training dataset.


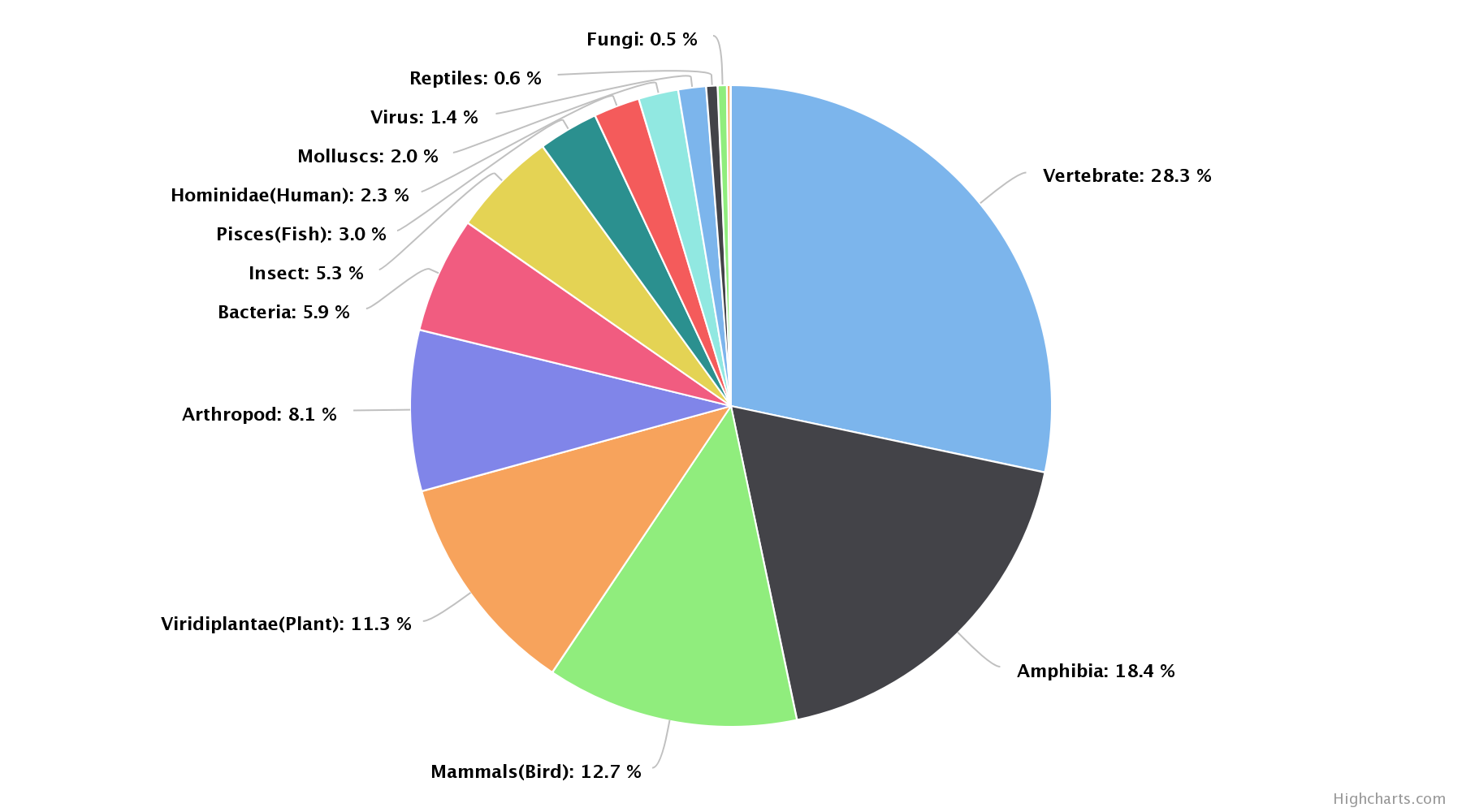


**Figure S3. The distribution of AMP source organisms in dbAMP.**


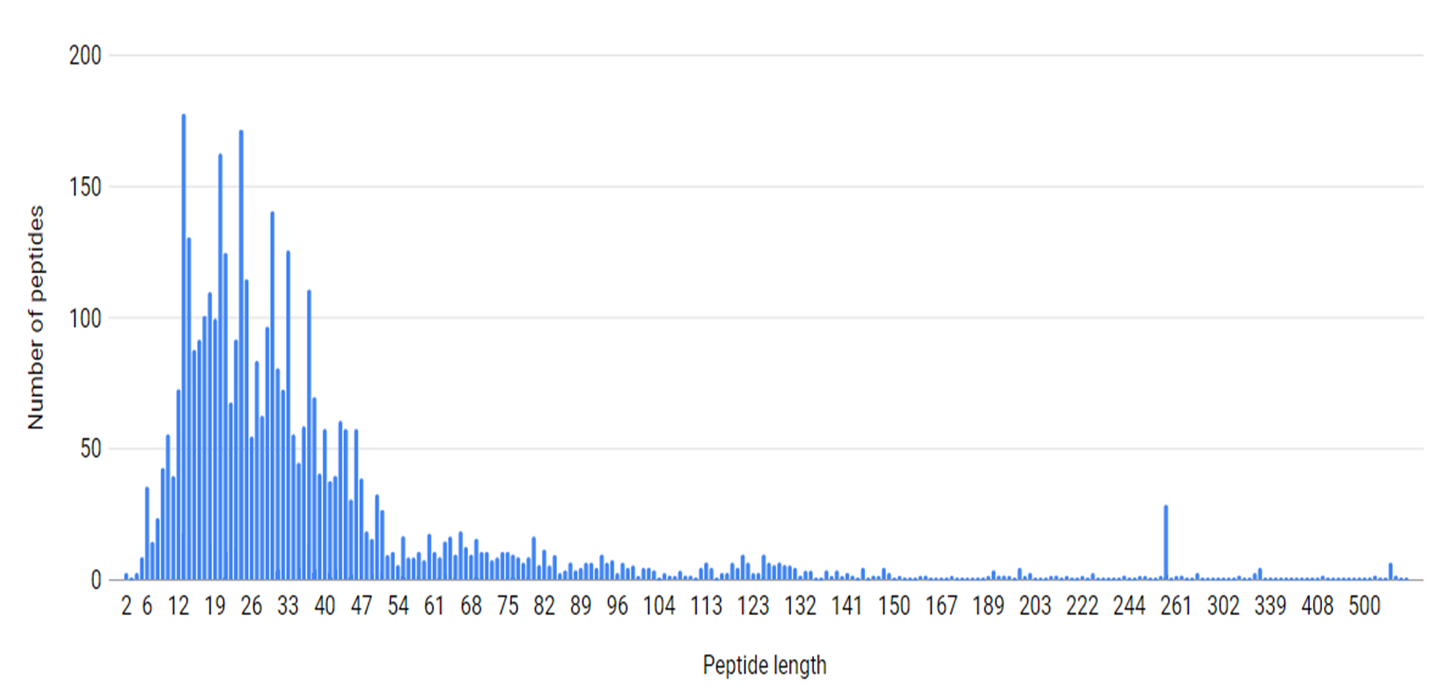


**Figure S4. Detailed distribution of peptide length of validated AMPs in dbAMP.**


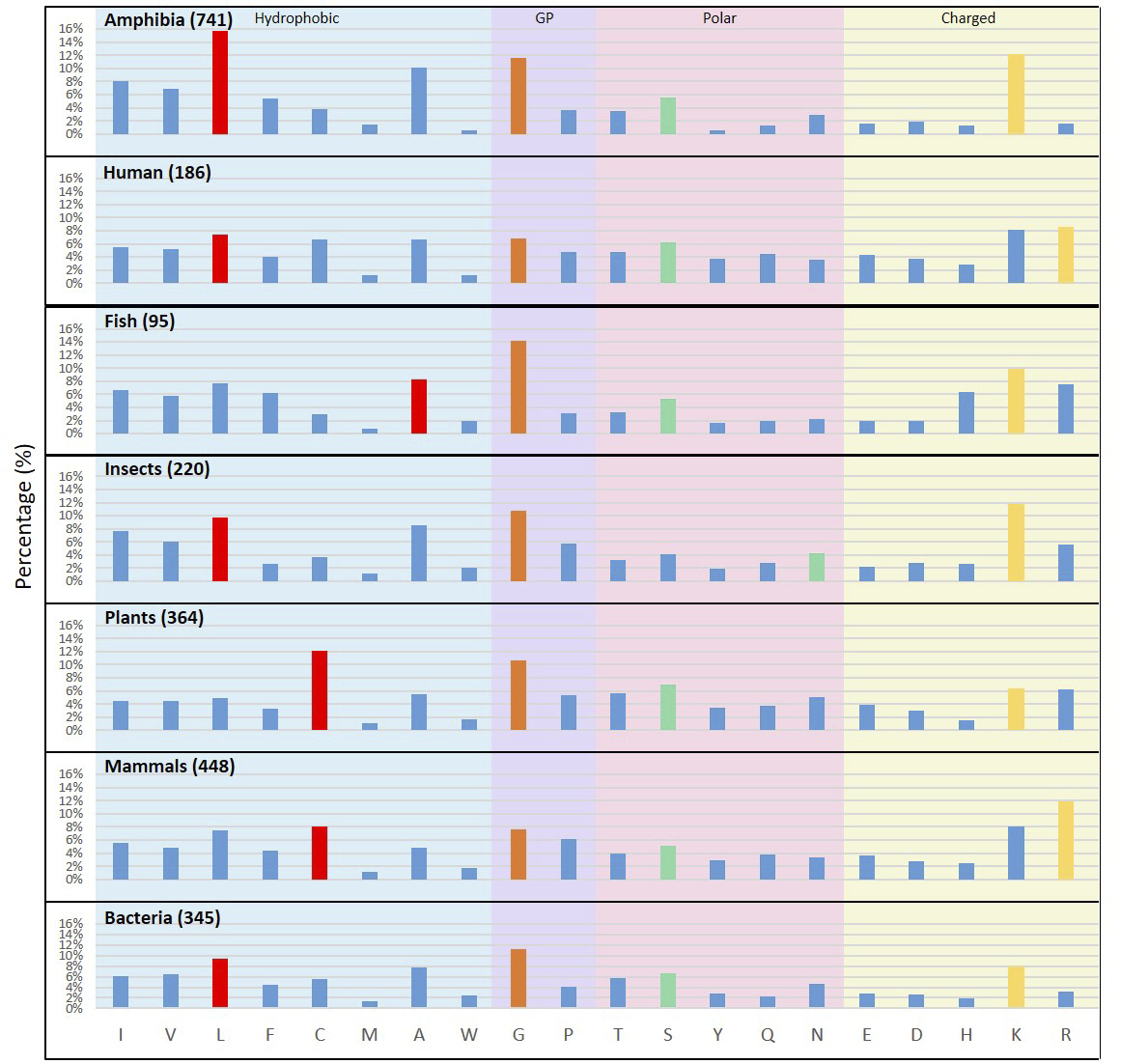


**Figure S5. Investigation of amino acid composition of AMPs based on different species.** The dominant amino acids (highest percentages) in the four amino acid groups (hydrophobic, nonpolar GP, polar and charged) are represented by highlighted color bars. This investigation indicated that there are noticeable differences of amino acid composition among these studied species. For instance, the L residue is significantly enriched in amphibia AMPs (16%), when comparing to that in other organisms with an average of 9.6%. The C residue is the most abundant amino acid in plant AMPs, which suggests that disulfide bonds are usual for rendering the peptide structure more stable**.** This abundance could be regarded as a very useful attribute for computational identification. Additionally, the frequencies of positively charged residues (K, R) are much higher in all organisms; meanwhile the negatively charged amino acids (E, D) are noticeably depleted. Interestingly, the cationic K residue is preferred over R residue in all organisms. These observations demonstrate that the information of positive net charge and hydrophobicity play a tremendous role in determining antimicrobial activity. Moreover, the G residue shows an overall abundance among all the seven life domains, owing to the frequent existence of glycine-rich AMPs in a wide variety of organisms. In contrast, Met (M) is rarely used (< 2%). This analysis indicates that the amino acid composition can play an important role in terms of evolution, structure, and function of natural AMPs.

**
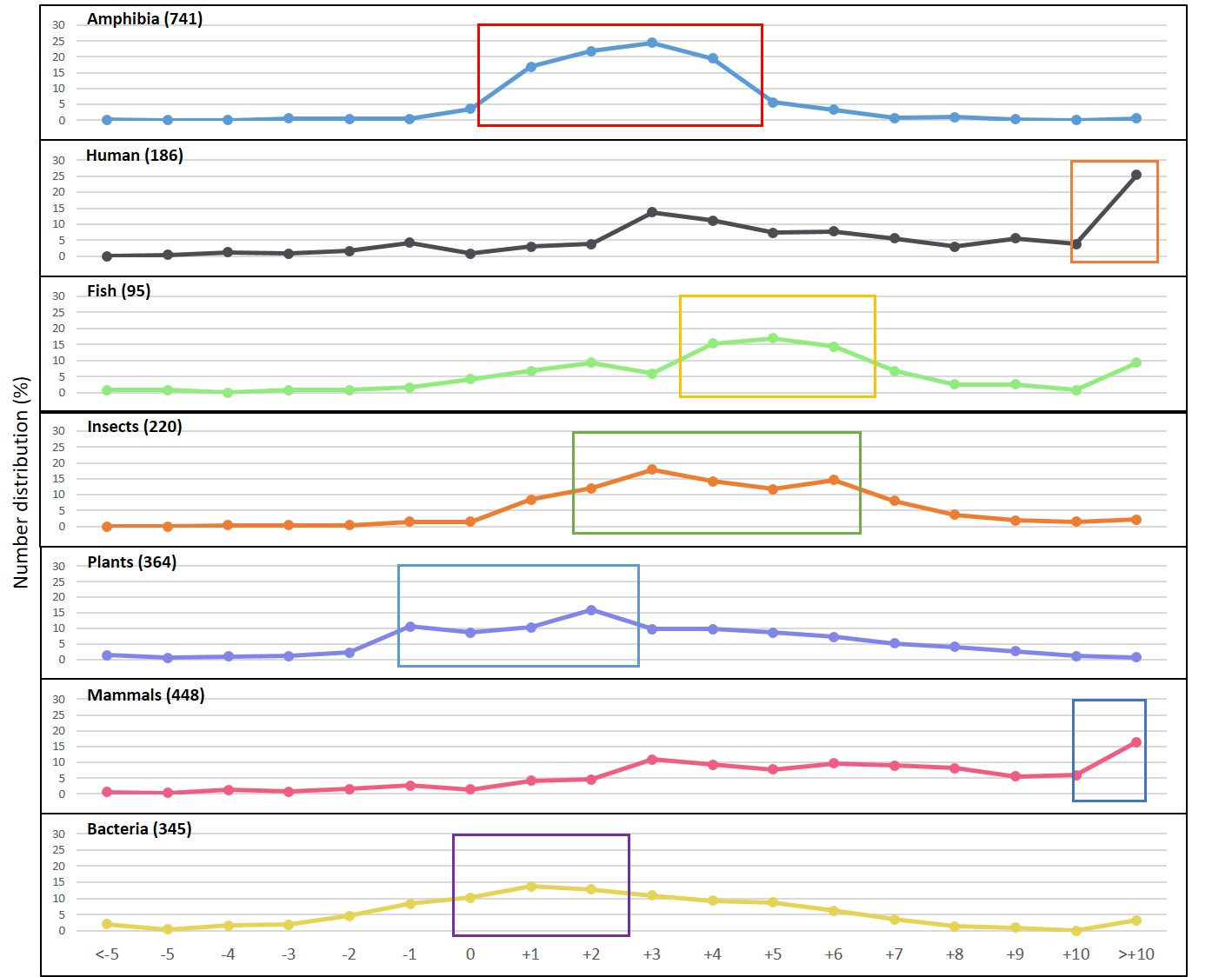
**

**Figure S6. Investigation of net charge distribution of AMPs based on different species.**


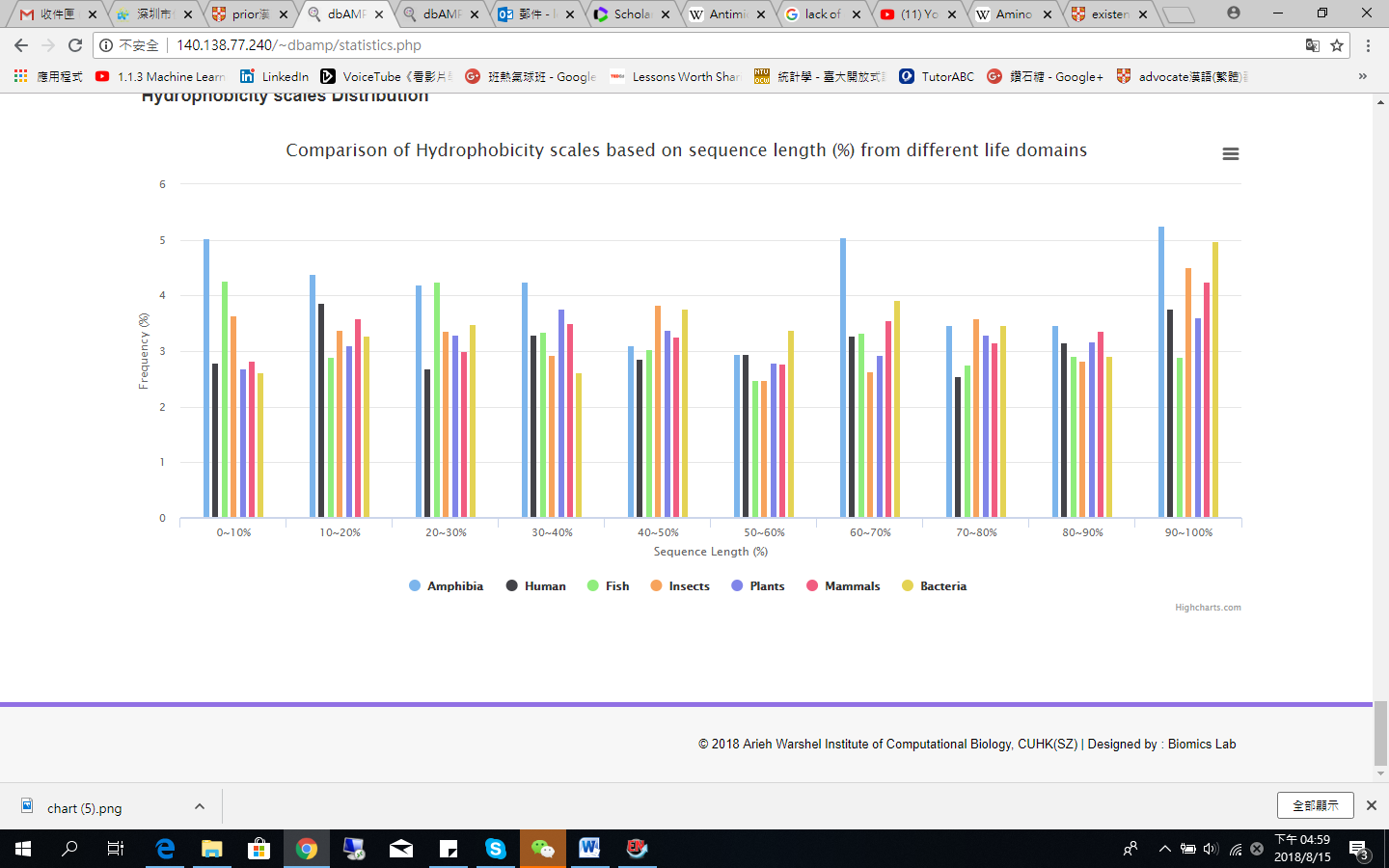


**Figure S7. Compositions of hydrophobic residues on different regions of AMPs based on their source species.**

**
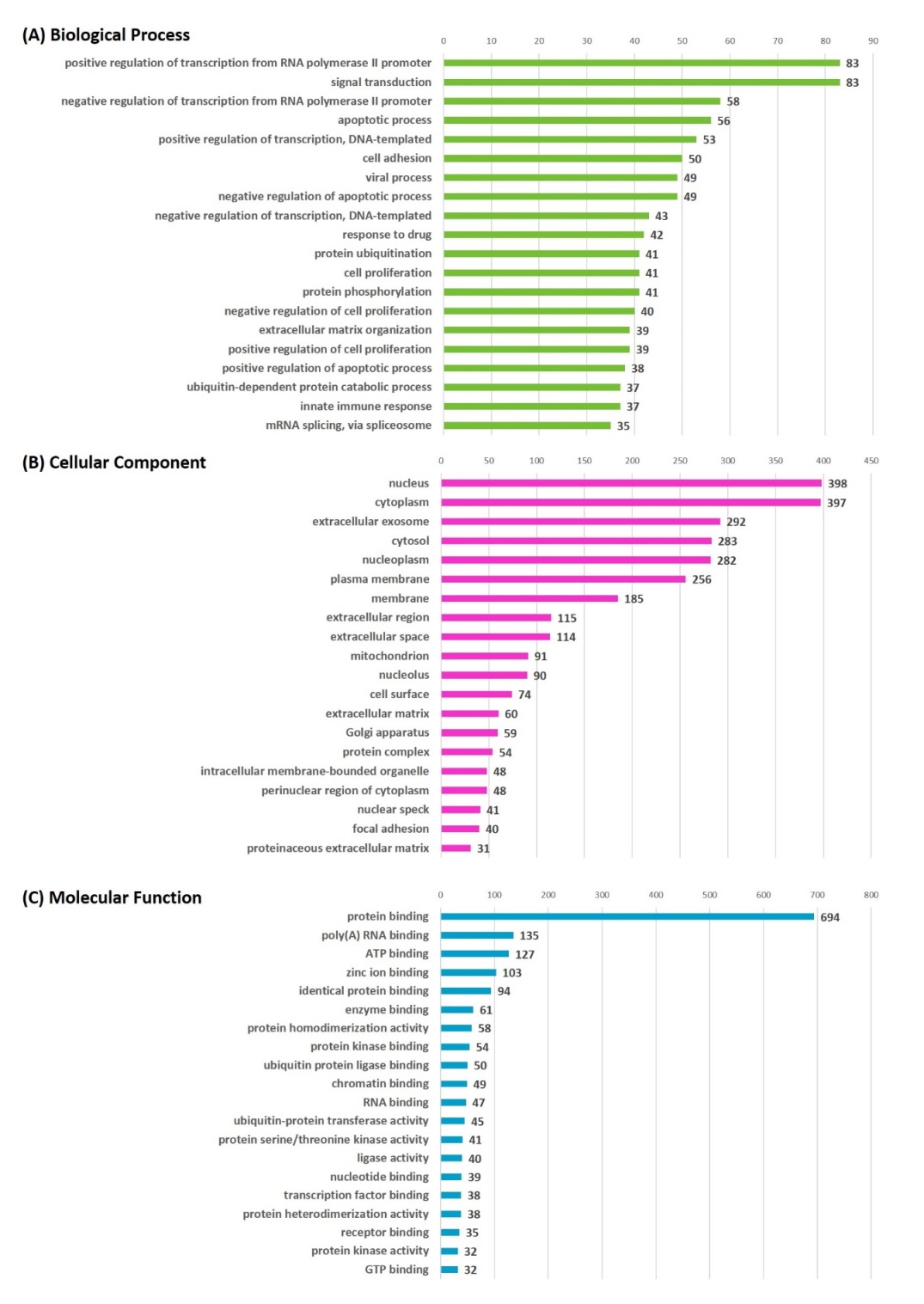
**

**Figure S8. Distribution of top 20 GO terms for AMP-interacting proteins.** The permeabilization of cell membranes was thought to be the primary killing mechanism of AMPs. However, there is an increasing evidence to prove that some AMPs can exert intracellular inhibitory activities as the primary or supportive mechanisms to achieve efficient killing procedure, but not damage the cell membrane. For instance, the AMP PR-39 obtained from pig intestines can defeat bacteria through suppressing proteins and DNA synthesis, while the human antimicrobial peptide LL-37 can migrate into the nucleus and co-localize with genomic DNA. This functional analysis revealed that the potential AMP-targeting protein tends to be affected in terms of nucleotide metabolism and signaling pathways with response to regulations of signal transduction, cell adhesion, viral infection and apoptotic process.


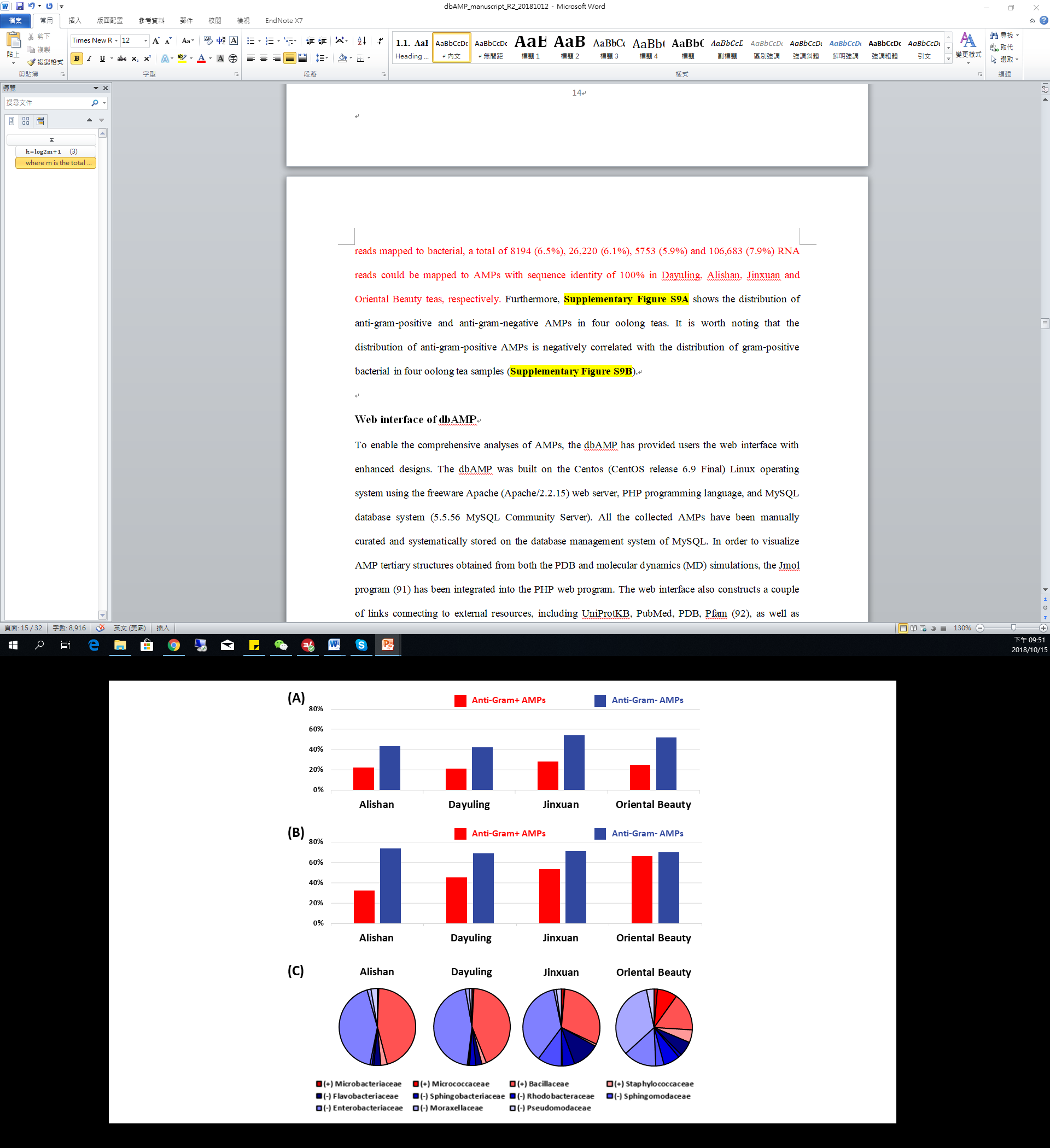


**Figure S9. Distribution of AMPs in four oolong teas. (A) The distribution of anti-gram-positive and anti-gram-negative AMPs in plants. (B) The distribution of anti-gram-positive and anti-gram-negative AMPs in bacterial. (C) The distribution of gram-positive and gram-negative bacterial in four oolong teas.**

**Table S1. Data statistics of integrated protein-protein interaction databases.** In addition to physical interactions, the STRING database also comprised a large-scale dataset of functional associations, including co-occurrence in literature abstracts, co-regulation in curated pathway, mRNA co-expression, etc., based on the specified confidence scores between proteins. In this investigation, a public network visualization software, Cytoscape, was integrated into dbAMP for displaying the interactions between AMP and proteins.

| **Database** | **Data source** | **Version** | **Number of proteins** | **Number of interactions** |
| --- | --- | --- | --- | --- |
| DIP | Physical interaction | 2012-02-28 | 25,996 | 76,147 |
| MINT | Physical interaction | 2012-08-01 | 35,553 | 241,458 |
| IntAct | Physical interaction | 2012-08-17 | 70,768 | 333,105 |
| APID | Physical interaction | 2012-09-01 | 56,460 | 322,579 |
| HPRD | Physical interaction | Release 9.0 | 30,047 | 41,327 |
| Interoporc | Physical interaction | 2008-09-29 | 44,313 | 206,337 |
| iRefIndex | Physical interaction | 2008-09-30 | 65,383 | 545,806 |
| UniProt | Physical interaction and functional association | 2013-07-24 | 4,011 | 6,941 |
| Reactome | Physical interaction and Pathway information | 2013-6-18 | 93,660 | 62,202 |
| Spike | Physical interaction and Pathway information | 2010-8-10 | 8,077 | 33,007 |
| STRING | Physical interaction and functional association | Release 9.0 | 5,214,234 | 224,346,017 |

**Table S2: Data statistics of training and testing datasets used for constructing and evaluating the predictive models, respectively, on seven species.**

| **Source species** | **Training dataset** | | **Testing dataset** | |
| --- | --- | --- | --- | --- |
|  | **Number of positive data** | **Number of negative data** | **Number of positive data** | **Number of negative data** |
| **Amphibia** | **741** | **1,595** | **185** | **398** |
| **Bacteria** | **345** | **6,040** | **86** | **1509** |
| **Fish** | **95** | **1,469** | **23** | **367** |
| **Human** | **186** | **6,595** | **46** | **1648** |
| **Insects** | **220** | **1,800** | **54** | **450** |
| **Mammals** | **448** | **6,919** | **111** | **1729** |
| **Plants** | **364** | **5,432** | **90** | **1358** |

**Table S3. Comparison of five-fold cross-validation results of four machine learning methods in different organisms.**

| **Amphibia** | | | | | |
| --- | --- | --- | --- | --- | --- |
| Classifier | Sn(%) | Sp(%) | Acc(%) | MCC | AUC(%) |
| Random forest | 99.19 | 99.18 | 99.19 | 0.981 | 99.19 |
| Decision tree | 97.84 | 98.81 | 98.50 | 0.965 | 98.33 |
| K-nearest neighbors | 96.76 | 99.81 | 98.84 | 0.973 | 98.29 |
| Support vector machine | 98.92 | 98.93 | 98.93 | 0.975 | 98.93 |
| **Bacteria** | | | | | |
| Classifier | Sn(%) | Sp(%) | Acc(%) | MCC | AUC(%) |
| Random forest | 95.94 | 96.18 | 96.16 | 0.735 | 96.06 |
| Decision tree | 86.67 | 97.95 | 97.34 | 0.769 | 92.31 |
| K-nearest neighbors | 73.62 | 99.44 | 98.04 | 0.7959 | 86.53 |
| Support vector machine | 95.94 | 95.94 | 95.94 | 0.725 | 95.94 |
| **Fish** | | | | | |
| Classifier | Sn(%) | Sp(%) | Acc(%) | MCC | AUC(%) |
| Random forest | 96.84 | 96.87 | 96.87 | 0.789 | 96.86 |
| Decision tree | 73.68 | 98.43 | 96.93 | 0.728 | 86.06 |
| K-nearest neighbors | 68.42 | 99.52 | 97.63 | 0.774 | 83.97 |
| Support vector machine | 82.11 | 99.86 | 98.79 | 0.889 | 90.99 |
| **Human** | | | | | |
| Classifier | Sn(%) | Sp(%) | Acc(%) | MCC | AUC(%) |
| Random forest | 94.09 | 93.07 | 93.10 | 0.489 | 93.58 |
| Decision tree | 74.19 | 98.15 | 97.49 | 0.615 | 86.17 |
| K-nearest neighbors | 68.28 | 98.94 | 98.10 | 0.654 | 83.61 |
| Support vector machine | 88.17 | 87.82 | 87.83 | 0.354 | 88.00 |
| **Insects** | | | | | |
| Classifier | Sn(%) | Sp(%) | Acc(%) | MCC | AUC(%) |
| Random forest | 96.36 | 96.33 | 96.34 | 0.838 | 96.35 |
| Decision tree | 91.36 | 97.56 | 96.88 | 0.849 | 94.46 |
| K-nearest neighbors | 85.91 | 98.28 | 96.93 | 0.842 | 92.10 |
| Support vector machine | 95.00 | 95.11 | 95.10 | 0.793 | 95.06 |
| **Mammals** | | | | | |
| Classifier | Sn(%) | Sp(%) | Acc(%) | MCC | AUC(%) |
| Random forest | 94.42 | 95.24 | 95.19 | 0.708 | 94.83 |
| Decision tree | 83.71 | 92.60 | 92.06 | 0.560 | 88.16 |
| K-nearest neighbors | 74.55 | 98.92 | 97.43 | 0.767 | 86.74 |
| Support vector machine | 93.97 | 93.97 | 93.97 | 0.662 | 93.97 |
| **Plants** | | | | | |
| Classifier | Sn(%) | Sp(%) | Acc(%) | MCC | AUC(%) |
| Random forest | 97.53 | 97.39 | 97.39 | 0.822 | 97.46 |
| Decision tree | 88.74 | 98.82 | 98.19 | 0.851 | 93.78 |
| K-nearest neighbors | 80.49 | 99.45 | 98.26 | 0.845 | 89.97 |
| Support vector machine | 96.70 | 96.70 | 96.70 | 0.786 | 96.70 |

**Table S4. Cross-validation and independet testing results of the generated RF models against multiple species.**

| **Life domain** | **Five-fold cross-validation**  **(Training dataset)** | | | | **Independent testing**  **(Testing dataset)** | | | |
| --- | --- | --- | --- | --- | --- | --- | --- | --- |
|  | **Sn (%)** | **Sp (%)** | **Acc (%)** | **MCC** | **Sn (%)** | **Sp (%)** | **Acc (%)** | **MCC** |
| Amphibia | 99.19 | 99.18 | 99.19 | 0.981 | 100.00% | 98.24% | 98.80 | 0.973 |
| Bacteria | 95.94 | 96.18 | 96.16 | 0.735 | 96.51% | 96.36% | 96.36 | 0.746 |
| Fish | 96.84 | 96.87 | 96.87 | 0.789 | 100.00% | 97.00% | 97.18 | 0.810 |
| Human | 94.09 | 93.07 | 93.10 | 0.489 | 97.83% | 92.17% | 92.33 | 0.482 |
| Insects | 96.36 | 96.33 | 96.34 | 0.838 | 100.00% | 97.56% | 97.82 | 0.900 |
| Mammals | 94.42 | 95.24 | 95.19 | 0.708 | 92.79% | 94.56% | 94.46 | 0.673 |
| Plants | 97.53 | 97.39 | 97.39 | 0.822 | 97.78% | 97.94% | 97.93 | 0.851 |

Abbreviation: Sn, sensitivity; Sp, specificity; Acc, accuracy; MCC, Matthews correlation coefficient.

**Table S5. Data statistics of RNA reads mapped to AMPs.** 150ng total RNA was used for RNA-seq library construction with Bacteria scriptseq complete kit (epicentre). Briefly, total RNA was first remove the ribosomal RNA. Then sequentially cDNA synthesize, 5’ tagging, 3’ tagging and index PCR to construct indexed library for Illumina sequencing platform. Libraries were qualification and quantification by Qubit and qPCR. After concentration adjustment, the libraries were mixed and denatured for sequencing. By applying the developed Docker container on the sequencing datasets, among the reads mapped to the plants, totally 1,077,775 (9.1%), 1,305,016 (6.8%), 1,152,178 (8.4%), and 366,454 (7.4%) RNA reads could be mapped to AMPs with sequence identity of 100% in Dayuling, Alishan, Jinxuan and Oriental Beauty teas, respectively. On the other hand, for the reads mapped to the bacterial, a total of 8194 (6.5%), 26,220 (6.2%), 5753 (6.1%) and 106,683 (7.7%) RNA reads could be mapped to AMPs with sequence identity of 100% in Dayuling, Alishan, Jinxuan and Oriental Beauty teas, respectively.

| **Teas** | | **Dayuling** | **Alishan** | **Jinxuan** | **Oriental Beauty** |
| --- | --- | --- | --- | --- | --- |
| **Number of raw RNA reads** | | 41,894,931 | 41,152,352 | 42,728,182 | 40,654,255 |
| **Number of reads after QC** | | 18,100,972 | 27,498,256 | 21,780,331 | 13,566,160 |
| **Plant** | **Number of reads mapped to plants** | 11,761,339 | 18,979,598 | 13,713,125 | 4,940,454 |
|  | **Number of reads mapped to AMPs** | 1,077,775(9.1%) | 1,305,016(6.8%) | 1,152,178(8.4%) | 366,454(7.4%) |
|  | **Number of AMPs** | 1,845 | 2,157 | 2,089 | 1,220 |
| **Bacterial** | **Number of reads mapped to bacterial** | 125,475 | 425,288 | 94,797 | 1,392,988 |
|  | **Number of reads mapped to AMPs** | 8,194(6.5%) | 26,220(6.2%) | 5,753(6.1%) | 106,683(7.7%) |
|  | **Number of AMPs** | 876 | 1,130 | 769 | 1,696 |
